# A combinatorial analysis using observational data identifies species that govern ecosystem functioning

**DOI:** 10.1371/journal.pone.0201135

**Published:** 2018-08-01

**Authors:** Benoît Jaillard, Philippe Deleporte, Michel Loreau, Cyrille Violle

**Affiliations:** 1 Ecologie fonctionnelle et Biogéochimie des Sols et Agrosystèmes, CIRAD, INRA, IRD, SupAgro, Univ Montpellier, Montpellier, France; 2 Centre for Biodiversity Theory and Modelling, Theoretical and Experimental Ecology Station, CNRS and Paul Sabatier University, Moulis, France; 3 Centre d’Ecologie Fonctionnelle et Evolutive, CNRS, Université de Montpellier, Université Paul-Valéry Montpellier, EPHE, Montpellier, France; Chinese Academy of Forestry, CHINA

## Abstract

Understanding the relationship between biodiversity and ecosystem functioning has so far resulted from two main approaches: the analysis of species' functional traits, and the analysis of species interaction networks. Here we propose a third approach, based on the association between combinations of species or of functional groups, which we term assembly motifs, and observed ecosystem functioning. Each assembly motif describes a biotic environment in which species interactions have particular effects on a given ecosystem function. Clustering species in functional groups generates a classification of ecosystems based on their assembly motif. We evaluate the quality of each species clustering, that is its ability to predict an ecosystem function, by the coefficient of determination of the ecosystem classification. An iterative process then enables identifying the species clustering in functional groups that best accounts for the functioning of the observed ecosystems. We test this approach using experimental and simulated datasets. We show that our combinatorial analysis makes it possible to identify the combinations of functional groups of species whose interactions govern ecosystem functioning without any a priori knowledge of the species themselves or their interactions. Our combinatorial approach reproduces the associative learning of empirical ecologists, and proves to be powerful and parsimonious.

## Introduction

Understanding the relationships between biodiversity and ecosystem functioning is of prime importance in ecology [1]. The analysis of these relationships has so far followed two main approaches: the analysis of the abundances and functional traits of species that make up the ecosystem (e.g. [2,3]), or the analysis of the network of species interactions, their connectivity, direction and intensity (e.g. [4,5]). In functional trait-based approaches, we need to know species’ properties. Moreover the effect induced by the species assemblage is expressed as a linear combination of the abundances and functional traits of each species [6–8]. The analysis of interaction networks is based on similar considerations: the direction and intensity of the effect of each interspecific interaction on ecosystem functioning have to be known a priori, and the overall effect of these interactions on ecosystem functioning is then assumed to be a combination of the various interaction-specific effects. Thus, both approaches require, on the one hand, detailed information about the elementary components of ecosystems, and on the other hand, an integrative process of these elementary contributions to infer overall ecosystem functioning. Although both approaches have proved their value, they fail to take into account the well-known non-linearity of most biological processes, in particular the highly non-linear way in which species interactions impact ecosystem functioning [9].

In nature, most people operate very differently. A walker, a hunter or a farmer records the identity of species they know, and associates each combination of species with a kind of ecosystem or with properties of the ecosystem [10,11]. For instance, they can easily associate tree species with a forest, brush species with a wasteland, and herbaceous species with a meadow. The ecosystem properties they observe depend on their interests: they might be land opening and accessibility by foot for the walker, fungal or hunting resources for the hunter, and soil fertility and biomass productivity for the farmer. These properties, however, always concern the ecosystem as a whole, not the species that make it up. This empirical approach to the relationship between biodiversity and ecosystem functioning requires learning about species and ecosystems, and its effectiveness depends on learning quality. Its predictive ability improves with observer’s experience, their knowledge of species and ecosystem responses to variations in biodiversity, and the accumulation of observations through field trips [12]. This associative approach takes into account the non-linearity of ecosystem responses to species composition, but it is descriptive and basically rests on repeated experiences gained by the observer.

Jaillard et al. [13] presented a combinatorial approach based on observed effects of species interactions on ecosystem functioning as a way to analyse the outcome of biodiversity-ecosystem functioning experiments. Their qualitative model was able to reproduce all the forms of biodiversity-ecosystem functioning relationships reported in the literature. More recently, Jaillard et al. [14] extended this approach by proposing an a posteriori method to cluster species based on their diversity effects, interaction and composition effects, on ecosystem functioning. This approach describes ecosystems as combinations of species' functional groups, called *assembly motifs* to echo network motifs in network theory [15]. The method requires a priori knowledge on the performance in monoculture of all species that make up the ecosystem. In this approach, another prerequisite is that any assembly motif is represented by more than one observed ecosystem. However, in nature, monocultures are exceptional, and most species combinations are unique. This currently restricts the application of the combinatorial approach to controlled experimental systems where the number of species is counted, each species needs to be grown in monoculture, and all species combinations needs to be assembled and grown.

Here we extend such a combinatorial approach to in situ situations where the sampling design is very sparse and incomplete, and where the performances in monoculture of each species are not known. We show how the functional structure of an ecosystem, that is the species whose interactions govern ecosystem functioning, can be determined by relying solely on its observed species composition. This is based on a procedure that clusters species into functional groups based on their effects on an observed ecosystem function and for which the fit to the observed data is maximised. More precisely, the procedure is based on an iterative process that improves step-by-step a criterion of convergence. The criterion of convergence is the coefficient of determination of the species clustering used as a criterion of model quality by Jaillard et al. [14]. Overall, the procedure builds a hierarchical divisive tree of clustering of species into functional groups that best accounts for observed ecosystem function. The clustering tree makes it possible to model the performance of all ecosystems, because it defines at least one hierarchical level where an ecosystem shares with others the same assembly motif. The modelling of the performance of all the ecosystems makes it possible to determine the optimal number of functional groups using the Akaike criterion, by comparing the different hierarchical levels of the species clustering tree. We test this approach using two observed datasets: a microbial diversity experiment [16] and the Cedar Creek Biodiversity II experiment [17] where we intentionally did not use the monoculture data in purpose. We evaluate the accuracy, sensitivity and repeatability of the combinatorial approach using virtual datasets. Virtual datasets are built on the basis of the two observed datasets, but assuming an idiosyncratic response of ecosystems to diversity. We show that our combinatorial approach determines the hidden structure in species' functional groups in all cases, provided that the sampling effort is sufficient. We therefore demonstrate that a combinatorial analysis of species makes it possible to identify, in experimental and field conditions, reliable functional groups of species whose interactions govern the ecosystem functioning.

## Material and methods

### Modelling the functioning of an ecosystem based on its species composition

**Fig 1:**
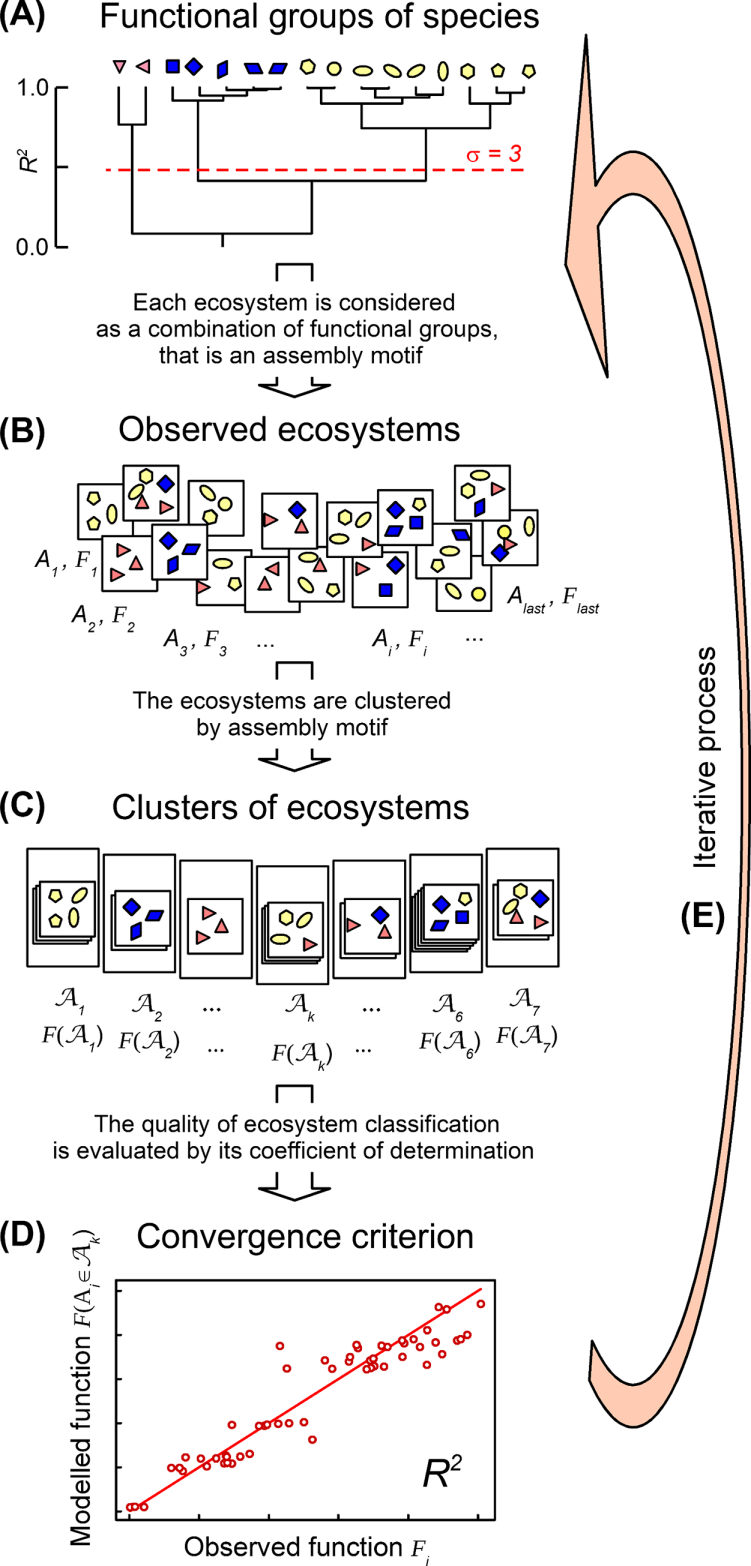
Step-by-step process to a posteriori cluster the species in functional groups based on an ecosystem function. (A) Species are a priori clustered in functional groups (here σ = 3 as an example). (B) Each ecosystem is then described by a combination of functional groups of species, that is an assembly motif (here *m =* 2^σ^-1 = 7 possible assembly motifs). (C) Ecosystems described by the same assembly motif are grouped in a given ecosystem clusters $A_{k}$ (*k* = 1 …7). (D) The goodness-of-fit of ecosystem classification is evaluated by its residual sum of squares (*RSS*_*modelling*_), that can be expressed as a coefficient of determination *R*^*2*^. The ecosystem classification is compared with others, rejected if worse but selected if better than others. (E) The process is iteratively repeated as long as a species clustering can generate an ecosystem classification better than all others. The process a posteriori selects the species clustering that best accounts for the observed ecosystem functioning.

The combinatorial model was presented in Jaillard et al. [14]. We consider a sample A of *n* ecosystems *A* observed in the field. Each ecosystem *A* is a combination of individuals that belong to different species *i*. We cluster the *s* species observed in the field into σ functional groups (Fig 1A). Each ecosystem can then be described as a combination of individuals that belong to different functional groups *j* of species (Fig 1B). We term *assembly motif* a combination of functional groups. The σ functional groups can be combined in 2^σ^-1 assembly motifs *M*_*k*_, out of which *m* are observed in the field (then *k =* 1,…,*m*). We associate an assembly motif *M*_*k*_ to each ecosystem *A* by assuming that each functional group of *M*_*k*_ is represented by at least one individual of species *i* in *A*, and that each species *i* of *A* belongs to a functional group of *M*_*k*_. Then, we define $A_{k}$ the cluster of ecosystems *A* described by the assembly motif *M*_*k*_ (Fig 1C). We define $A_{i,k}$ (with *i =* 1,…,*s* and *k =* 1,…,*m*) the cluster of ecosystems *A* of $A_{k}$ that contains at least one individual of the species *i*. Therefore, ecosystems of $A_{i,k}$ share the same assembly motif and contain the same species.

For each ecosystem *A* of A, a function *F*_*observed*_(*A*), an ecosystem property such as biomass production, respiration or nutrient recycling, is observed. The function of each ecosystem results from so-called diversity effects, i.e. the composition of species and interactions between species within the ecosystem. We postulate that diversity effects are recapitulated by an assembly motif, that is by a functional composition [18]. As a consequence, we can simply model the function *F*_*modelled*_(*A)* of any ecosystem *A* associated to the assembly motif *M*_*k*_ and that contains the species *i*, as the average of mean functions of ecosystem clusters $A_{i,k}$ of species *i* of *A*, i.e. ecosystems that share the same assembly motif and contain the same species than *A*:

$$F_{modelled}(A\in A_{k})=\bar{\underset{i\in A}{F}}(A_{i,k}).$$

### Evaluating the accuracy of a species clustering

The function of observed ecosystems is statistically characterized by its mean $F_{observed}(A\in A)$ and its variance. The variance is proportional to the sum of squares of deviations from the mean, the total sum of squares TSS(A):

$$TSS(A)=\sum_{A\in A}{\left(F_{observed}(A)-{\bar{F}}_{observed}(A)\right)}^{2}.$$

Each species clustering by σ functional groups is a model (Fig 1A). Each σ-model generates an ecosystem classification into *m* assembly motifs (*m* increases with σ) (Fig 1B and 1C). This classification further allows to estimate the function *F*_*modelled*_(σ,*A*) of each ecosystem. The goodness of fit of the σ-model is evaluated by the sum of squares of deviations between the observed function and modelled function, that is the residual sum of squares $RSS_{modelling}(\sigma ,A)$ [19] (Fig 1D):

$${RSS}_{modelling}(\sigma ,A)=\sum_{A\in A}{\left(F_{observed}(A)-F_{modelled}(\sigma ,A)\right)}^{2}.$$

The coefficient of determination, noted $R^{2}(\sigma ,A)$, is defined as the variance proportion explained by the σ-model. $R^{2}(\sigma ,A)$ is thus comprised between 0 and 1. $R^{2}(\sigma ,A)$ can also be expressed as the ratio between the residual sum of squares $RSS_{modelling}(\sigma ,A)$ and the total sum of squares TSS(A), subtracted from 1 (Fig 1D):

$$R^{2}(\sigma ,A)=1-\frac{{RSS}_{modelling}(\sigma ,A)}{TSS(A)}.$$

$R^{2}(\sigma ,A)$ evaluates the explanatory ability of a σ-model of species clustering by σ functional groups. For each given number σ of functional groups, we can then select the σ-model that best accounts for the observed ecosystem functioning (Fig 1E).

### Evaluating the predictive ability of a species clustering

The coefficient of determination increases with the number of functional groups until reaching one when there are as many functional groups as species. Alone, the coefficient of determination does not measure the quality of the model: it must be completed by the evaluation of its predictive ability. The predictive ability of the σ-model is evaluated by cross-validation [19]. The predicting error is equal to the sum of squares of deviations between the observed and independently predicted function, that is the residual sum of squares $RSS_{predicting}(\sigma ,A)$:

$${RSS}_{predicting}(\sigma ,A)=\sum_{A\in A}{\left(F_{observed}(A)-F_{predicted}(\sigma ,A)\right)}^{2}.$$

As the goodness of fit, the predicting error can be divided by the total sum of squares TSS(A) and expressed as the efficiency of the σ-model, noted *E* [20]:

$$E(\sigma ,A)=1-\frac{{RSS}_{predicting}(\sigma ,A)}{TSS(A)}.$$

The efficiency E(σ,A) can be lower than zero (*E*∈[-∞,1]), the model can be a worse predictor than the mean of observed functions. $R^{2}(\sigma ,A)$ and E(σ,A) evaluate the quality of a σ-model of species clustering by σ functional groups: the higher they are, the better the explanatory and predictive abilities of the model.

### Building a hierarchical divisive tree of species clustering

When an ecosystem is the sole representative of its motif, the function of this ecosystem cannot be independently predicted. The number of ecosystems whose function cannot be independently predicted increases with the number of functional groups, because the number of ecosystems associated to each assembly motif decreases when the number of functional groups increases. It follows that, for different species clustering by functional groups, and different numbers σ of functional groups, the σ-models refer to different numbers of ecosystems: this prohibits to compare different σ-models. To make sure that different σ-models of species clustering explain and predict the function of all ecosystems, we build a hierarchical divisive tree of species clustering. Indeed, the function of any ecosystem is, at least, predicted by the mean function of all ecosystems when σ = 1, that is when all species are redundant, then there is a unique functional group, and all ecosystems share the same, unique and trivial assembly motif.

We adapt a hierarchical clustering algorithm classically used to cluster objects [21]. We use the coefficient of determination $R^{2}(\sigma ,A)$ of σ-models as a convergence criterion. We proceed by division, varying the number of functional groups of species from 1 to *s*, that is the total number of species (Fig 1). All species are initially regrouped into a single large functional group (σ = 1). At each step, one of the functional groups is split into two new functional groups: the new functional groups selected are those that maximize $R^{2}(\sigma ,A)$ of the σ-models. The process stops when each species is isolated in a singleton (σ = *s*). As a whole, the process generates a hierarchical divisive tree of species clustering, whose $R^{2}(\sigma ,A)$ increases monotonically with the number of functional groups (Fig 1A).

At each hierarchical level of the divisive tree, the division of the σ existing functional groups into new σ+1 functional groups proceeds as follows. Each existing functional group is successively split into two new functional groups. To do that, each species of the functional group is isolated into a singleton: the singleton-species that maximizes $R^{2}(\sigma +1,A)$ is selected as the nucleus of the new functional group. Each of the other species belonging to the existing functional group is successively moved towards the new functional group: the species clustering that maximizes $R^{2}(\sigma +1,A)$ is kept. Moving species into the new functional group continues as long as the new species clustering, that is the (σ+1)-model, increases $R^{2}(\sigma +1,A)$. When each σ existing functional group has been split, there are σ (σ+1)-model candidates as a maximum: the (σ+1)-model that maximizes $R^{2}(\sigma +1,A)$ is kept.

At the first hierarchical level (σ = 1), the split into two functional groups of the initial single large functional group that contains *s* species needs to evaluate at the most *s*+(*s*-1)+(*s*-2)+…+2 = *s*(*s*+1)/2-1 species clustering. The building of the whole divisive tree of species clustering therefore needs to evaluate at the most [*s*(*s*+1)/2-1]+[(*s-*1)*s*/2-1]+…+2 = *s*[(*s*+1)(*s*+2)/6-1] species clustering.

When the hierarchical tree is built, the efficiency E(σ,A) of the σ-models that best account for the ecosystem function is determined (σ∈[1,…,*s*]). Contrary to $R^{2}(\sigma ,A)$, E(σ,A) can increase or decrease with the number of functional groups. A decrease in E(σ,A) means that the corresponding σ-model is a worse predictor than the previous model of σ-1 functional groups. In this case, the σ-model is rejected, as well as all subsequent splits of functional groups: in other words, only the base of the hierarchical tree, from 1 to σ-1 functional groups, is considered. The efficiency of selected models thus necessarily increases monotonically with the number of functional groups until reaching a maximum at σ_*max*_ = σ-1 functional groups.

As the number of functional groups increases, the predictability of the function of each ecosystem increases until the ecosystem is the sole representative of its assembly motif: then its function can no longer be predicted. We consider the last prediction as the best possible one for this ecosystem, and we denote by σ*'*(*A*) the highest number of functional groups which allows to independently predict the function of this ecosystem:

$$\sigma \prime (A)=\underset{j\in [1,\ldots ,\sigma ]}{argmax}\;\left(F_{predicted}\left(j,A\right)\right).$$

### Evaluating the quality of a hierarchical tree of species clustering

Next, we define two quality criteria for the whole hierarchical tree. The sum of squares of the deviations between observed and modelled function $RSS_{tree,modelling}(\sigma ,A)$ on the one hand, and the sum of squares of the deviations between observed and predicted functions $RSS_{tree,predicting}(\sigma ,A)$ on the other hand, are then expressed as follows:

$${RSS}_{tree,modelling}(\sigma ,A)=\sum_{A\in A}{\left(F_{observed}(A)-F_{modelled}(\sigma \prime (A),A)\right)}^{2},$$

and:

$${RSS}_{tree,predicting}(\sigma ,A)=\sum_{A\in A}{\left(F_{observed}(A)-F_{predicted}(\sigma \prime (A),A)\right)}^{2},$$

respectively. The tree coefficient of determination $R^{2}{}_{tree}(\sigma ,A)$, from its base to the level of species clustering into σ functional groups, and the tree efficiency $E_{tree}(\sigma ,A)$ are then written:

$$R_{tree}^{2}(\sigma ,A)=1-\frac{{RSS}_{tree,modelling}(\sigma ,A)}{TSS(A)},$$

and:

$$E_{tree}(\sigma ,A)=1-\frac{{RSS}_{tree,predicting}(\sigma ,A)}{TSS(A)},$$

respectively. $R^{2}{}_{tree}(\sigma ,A)$ and $E_{tree}(\sigma ,A)$ are, by construction, lower or equal to $R^{2}(\sigma ,A)$ and E(σ,A), respectively. $R^{2}{}_{tree}(\sigma ,A)$ is comprised between 0 and 1, and $E_{tree}(\sigma ,A)$ can be lower than zero (*E*_*tree*_∈[-∞,1]). All along the hierarchical tree of species clustering, the two quality criteria refer to the same ecosystem sample and to the same number of observed, modelled and predicted ecosystems.

### Evaluating the optimum number of functional groups

We compare the species clustering by different numbers of functional groups along a hierarchical tree. We use the Akaïke's criterion, corrected for the second order bias [22,23]. This criterion comprises two terms: model likelihood and model parametrization. The model likelihood refers to the number of observed ecosystems, *n*. The model parametrization refers to the number *m* of assembly motifs that are observed in the field. The Akaïke's criterion $AICc_{tree}(\sigma ,A)$ for a clustering tree is thus written as:

$${AICc}_{tree}(\sigma ,A)=nlog\left(\frac{{RSS}_{tree,modelling}(\sigma ,A)}{n}\right)+2m+\frac{2m(m+1)}{\left(n-m+1\right)}.$$

The lowest value of $AICc_{tree}(\sigma ,A)$ indicates the optimum number σ"(A) of functional groups: it results from a trade-off between model accuracy and model parsimony:

$$\sigma "(A)=\underset{\sigma \in [1,\ldots ,s)}{argmin}({AICc}_{tree}(\sigma ,A)).$$

### Observed biodiversity datasets

To test our approach we use two observed datasets, already used in Jaillard et al. [14]. The first dataset is based on the observation of all 2^6^−1 = 63 bacteria combinations assembled from an initial pool of 6 species. Langenheder et al. [16] designed this experiment to analyse the biodiversity-productivity relationship in bacterial microcosms. We analyse the xylose oxidation (ecosystem function) after 48 hours for the 57 pluri-species bacterial ecosystems.

The second dataset is from the Cedar Creek Biodiversity II experiment of Tilman et al. [17]. This experiment was devoted to the analysis of the biodiversity-productivity relationship in grasslands. The observed ecosystem function is the annual aboveground biomass per unit area. The experiment contains 88 different ecosystems assembled from 16 grassland species, of which 53 are pluri-species ecosystems. The 16 grassland species were a priori grouped into 4 functional groups of 4 species: legumes, forbs, C3- and C4-grasses.

### Virtual biodiversity datasets

We test the accuracy and robustness of the combinatorial approach using virtual datasets that mimic the two observed datasets. The datasets are built around an a priori functional structure. The species are individually named, and clustered in functional groups. All possible combinations of species, that is all possible ecosystems, are assembled. The combinations of functional groups determine a set of assembly motifs: each ecosystem is associated to an assembly motif according to its species composition.

Our model assumes that ecosystems *A* that share the same assembly motif *M*_*k*_ display a similar function. We assume that the function $F(A\in A_{k})$ of each ecosystem is equal to the mean function $F(A_{k})$ of ecosystems that share its assembly motif, with a random error $\varepsilon (A\in A_{k})$. The mean functions $F(A_{k})$ of ecosystems that share the same assembly motif are randomly drawn inside an interval of values, according to a uniform law. The error $\varepsilon (A\in A_{k})$ follows a standard normal distribution, truncated at ±2 to avoid negative values and with mean standard deviation adjusted to 1. It is assumed to be proportional to $F(A_{k})$ and the mean relative error *c*_*v*_ (assumed constant for all assembly motifs), then:

$$F(A\in A_{k})=\bar{F}(A_{k})+\varepsilon (A\in A_{k}),$$

with:

$$\varepsilon (A\in A_{k})∼\bar{F}(A_{k})N(0,1)c_{v}.$$

This virtual ecology exercise helps us to answer three questions. The first question concerns the accuracy and repeatability of the combinatorial approach, the accuracy of the determination of the optimum number of functional groups using the Akaïke index, and finally the reliability of results obtained on observed datasets. The aim is to verify that a combinatorial analysis allows to accurately identify an a priori functional structure (number and size of functional groups), and the identity of species that belong to the functional groups. For this, we use the Jaccard index, which is equal to one when the solution is right. We mimic both the Langenheder's [16] and Biodiversity II [17] experiments, by building datasets whose functional structure includes a given number of functional groups, varying between 2 and *s*-1 functional groups from a simulation to another. To fix the species clustering, we use the results obtained on the observed datasets. The combinatorial analysis generates hierarchical trees of species clustering: each tree is used to define a priori functional structure of ecosystems, with 2 to 5 functional groups in the Langenheder's experiment [16], with 2 to 15 functional groups in the Biodiversity II experiment [17].

We evaluate the accuracy of the combinatorial approach versus the number σ of functional groups of species. In the microbial experiment, all 63 possible combinations were observed: we analyse all the ecosystems for each number of functional groups. The mean function of assembly motifs is drawn at random in the interval 0.4–1.4 xylose oxidation, and the relative error is fixed to 0.08. In the Biodiversity II experiment, 16 species allow to assemble 65 519 pluri-species ecosystems: we randomly sample and analyse 2048 ecosystems for each number of functional groups. The mean function of assembly motifs is drawn at random in the interval 50–550 g m^-2^ aboveground biomass, and the relative error is fixed to 0.17.

The second question concerns the sensitivity of the combinatorial approach to the relative error on ecosystem function. We use the Langenheder's experiment [16] where all possible species combinations were observed. The experiment is simulated using a set of 6 species grouped into 3 functional groups of size 1, 2 and 3 species, respectively. This leads to 7 possible assembly motifs whose mean functions are drawn at random in the interval 0.4–1.4 xylose oxidation. The relative error is assumed to be constant for each simulation, but it varies between 0.02 and 1.28 from a simulation to another.

The third question concerns the effect of sampling effort on the method accuracy. We use the Biodiversity II experiment [17] where only 53 pluri-species ecosystems were observed among the 65 519 possible pluri-species ecosystems. The experiment is simulated using a set of 16 species grouped into 4 functional groups of size 1, 2, 6 and 7 species, respectively. This leads to 15 possible assembly motifs whose mean functions are drawn at random in the interval 50–550 g m^-2^ of aboveground biomass. The relative error is assumed constant, equal to 0.17. The number of sampled ecosystems size varies between 32 and 2048 pluri-species ecosystems.

In each simulated experiment, after each random drawing of ecosystem sample, the mean function of each assembly motif and the function of each ecosystem are randomly generated to avoid any bias in the virtual datasets. Each ecosystem sampling and combinatorial analysis are repeated 100 times. All the computations are done using the R-software [24]. The scripts are available from the Dryad Digital Repository.

## Results

### Combinatorial analysis of ecosystem composition

**Fig 2:**
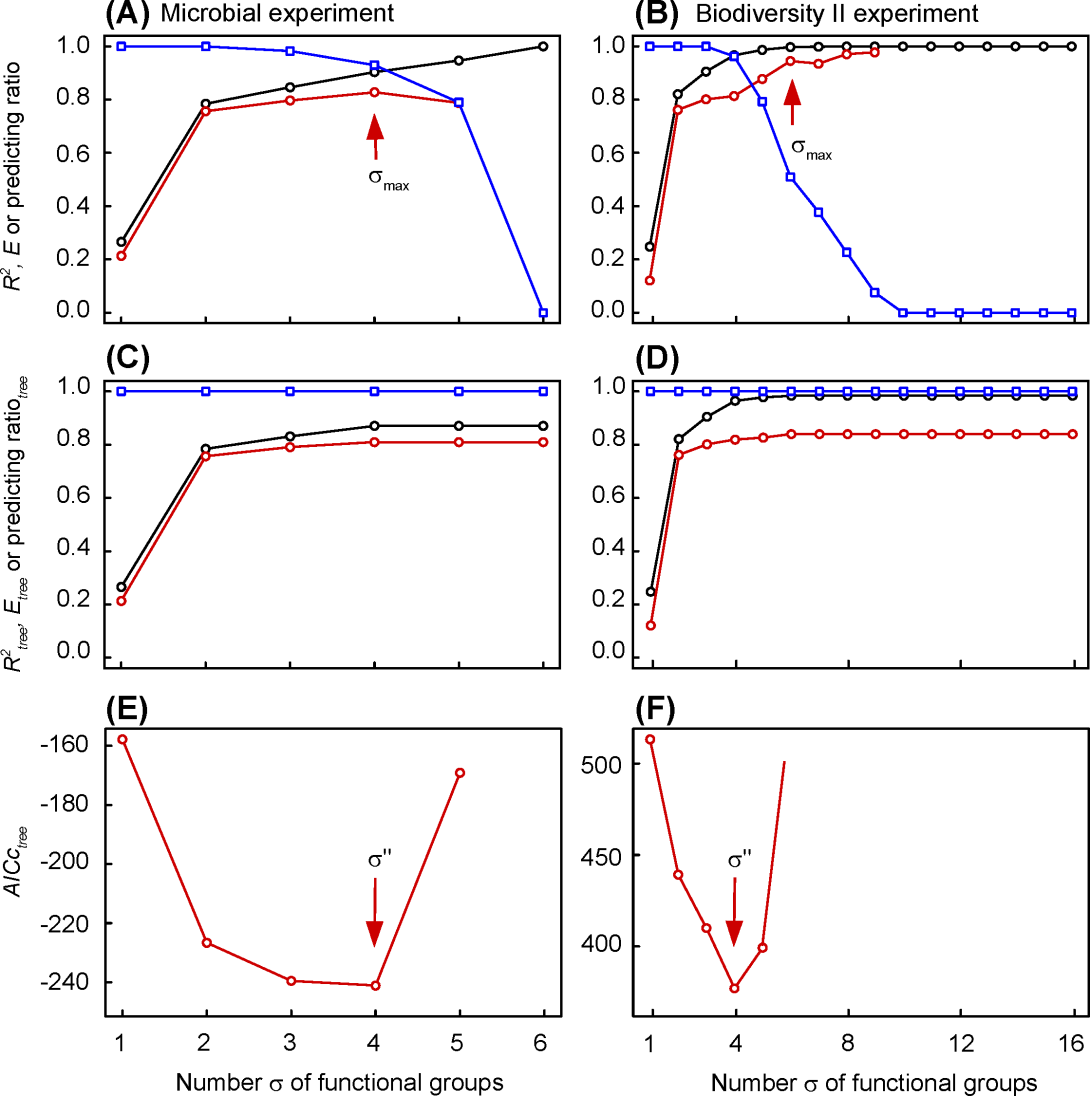
Changes in coefficient of determination, coefficient of efficiency, predicting ratio and Akaïke index versus the number of functional groups. (A), (C) and (E) Microbial experiment of Langenheder et al. [16]. (B), (D) and (F) Biodiversity II experiment of Tilman et al. [17]. (A) and (B) Coefficient of determination (black circle), coefficient of efficiency (red circle) and predicting ratio (blue square) of each clustering model. The arrow indicates the first local maximum of the coefficient of efficiency, located at σ_*max*_ functional groups. (C) and (D) Coefficient of determination (black circle), coefficient of efficiency (red circle) and predicting ratio (blue square) of whole tree of species clustering. (E) and (F) tree Akaïke index corrected for the second order bias (*AICc*). The arrow indicates the minimum value of Akaïke index, that corresponds to the optimum number σ" of functional groups.

The step-by-step species clustering improves the coefficient of determination of the resulting ecosystem classification: *R*^*2*^ increases from a low value when all the species are regrouped together in a single large functional group (σ = 1), to one when each species is isolated in a singleton (σ = *s*) (Fig 2A and 2B). The efficiency *E* increases up to a first local maximum (at σ_*max*_ = 4 in the microbial experiment and σ_*max*_ = 6 in the Biodiversity II experiment). Beyond this maximum, the increase in the number of functional groups does not improve the predictive ability of the clustering models: the leaves of the tree are therefore not validated. The predicting ratio, that is the proportion of ecosystems whose function can be predicted, decreases from 1 to 0 when the number of functional groups increases from 1 to *s*: it decreases slowly in the microbial experiment, more rapidly in the Biodiversity II experiment.

The efficiency *E*_*tree*_ and coefficient of determination *R*^*2*^_*tree*_ of hierarchical trees of species clustering increase monotonically with the number of functional groups, until a plateau is reached (Fig 2C and 2D). The plateau corresponds to the best explanatory and predictive capacities of the species clustering trees: *E*_*tree*_ and *R*^*2*^_*tree*_ are 0.839 and 0.896, respectively, in the microbial experiment, and 0.819 and 0.965, respectively, in the Biodiversity II experiment. These two criteria of tree quality correspond to a predicting ratio_*tree*_ of 1, that is the function of all ecosystems is predicted by the model (Fig 2C and 2D). The *AICc*_*tree*_ Akaïke index is minimum for σ" = 4 functional groups in both experiments (Fig 2E and 2F): σ" = 4 out of 6 species in the microbial experiment, but σ" = 4 out of 16 species in the Biodiversity II experiment.

**Fig 3:**
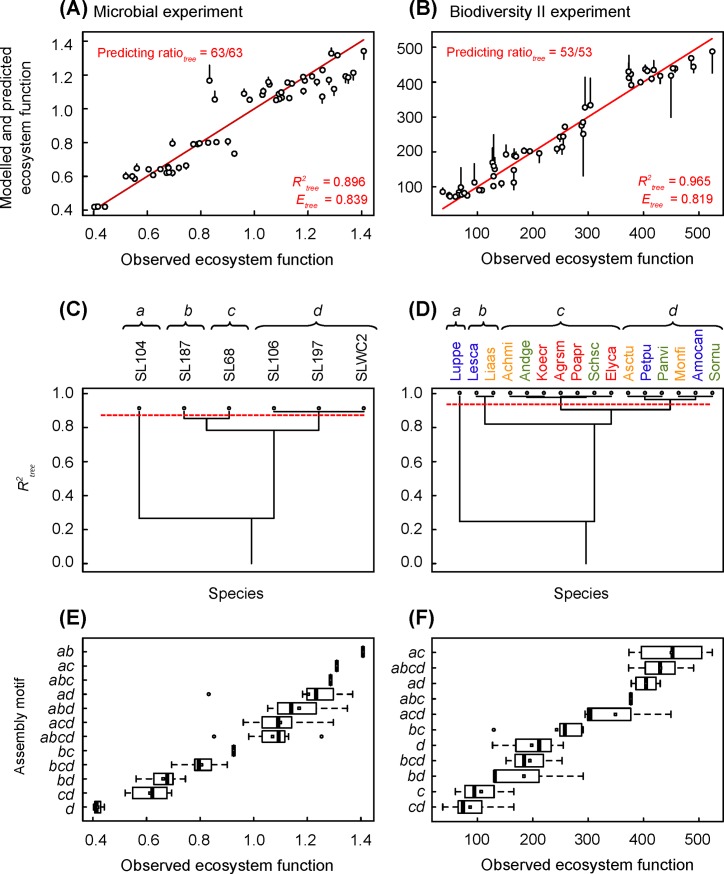
Species clustering, goodness-of-fit and predictive ability of the whole hierarchical trees of species clustering. (A), (C) and (E) Microbial experiment of Langenheder et al. [16]. (B), (D) and (F) Biodiversity II experiment of Tilman et al. [17]. (A) and (B) Modelling of ecosystem function based on the hierarchical tree of species clustering, from 1 to 4 functional groups of species in both experiments (see Fig 2E and 2F). Each bar corresponds to the error induced by leaving out the ecosystem to predict. Solid red line is the 1:1 line. (C) and (D) Hierarchical tree of species clustering. Dotted red line corresponds to the optimum number of functional groups indicated by *AICc*. Functional groups are noted by italic lowercase letters. (E) and (F) Boxplots of ecosystem functions by assembly motifs.

In the microbial experiment (Fig 3A, 3C and 3E), the species clustering explains 89.6% of the observed variance and its efficiency is 83.9% (*E*_*tree*_*/R*^*2*^_*tree*_ = 0.936). The species SL104 is first isolated in a singleton, then the five remaining species are split in two functional groups, one that brings together species SL187 and SL68, the other species SL106, SL197 and SLWC2. The combinatorial analysis finally allows to cluster the 6 species used by Langenheder et al. [16] in three effect functional groups: {SL104}, {SL187, SL68} and {SL106, SL197, SLWC2}. It suggests that the two species SL187 and SL68 induce significantly different effects and could be considered as two functional singletons (Fig 3C). This species clustering generates 15 assembly motifs, of which 12 are observed (Fig 3E): the ecosystem function does not vary with the number of functional groups. However, the functional group *a =* {SL104} occurs only in ecosystems with the highest function, and the functional group *d* occurs mainly in ecosystems with the lowest function (Fig 3E).

In the Biodiversity II experiment (Fig 3B, 3D and 3F), the species clustering accounts for 96.5% of the total variance, and its efficiency is 81.9% (*E*_*tree*_*/R*^*2*^_*tree*_ = 0.849). The species {*Lupinus perennis*} is first isolated in a singleton, then the species {*Lespedeza capitata*, *Liatris aspera*} in a doubleton. The 13 remaining species are finally split in two functional groups, one clustering species {*Asclepias tuberose*, *Petalostemum purpureum*, *Panicum virgatum*, *Monarda fistulosa*, *Amorpha canescens*, *Sorghastrum nutans*}, the other species {*Achillea millefolium*, *Andropogon gerardi*, *Koeleria cristata*, *Agropyron smithii*, *Poa pratensis*, *Schizachyrium scoparium*, *Elymus canadensis*}. This species clustering generates also 15 assembly motifs, of which 11 are observed: the ecosystem function does not vary with the number of functional groups, but the functional group *a =* {*Lupinus perennis*} occurs only in ecosystems with the highest function (Fig 3F).

### Accuracy of the combinatorial analysis of ecosystem composition

**Fig 4:**
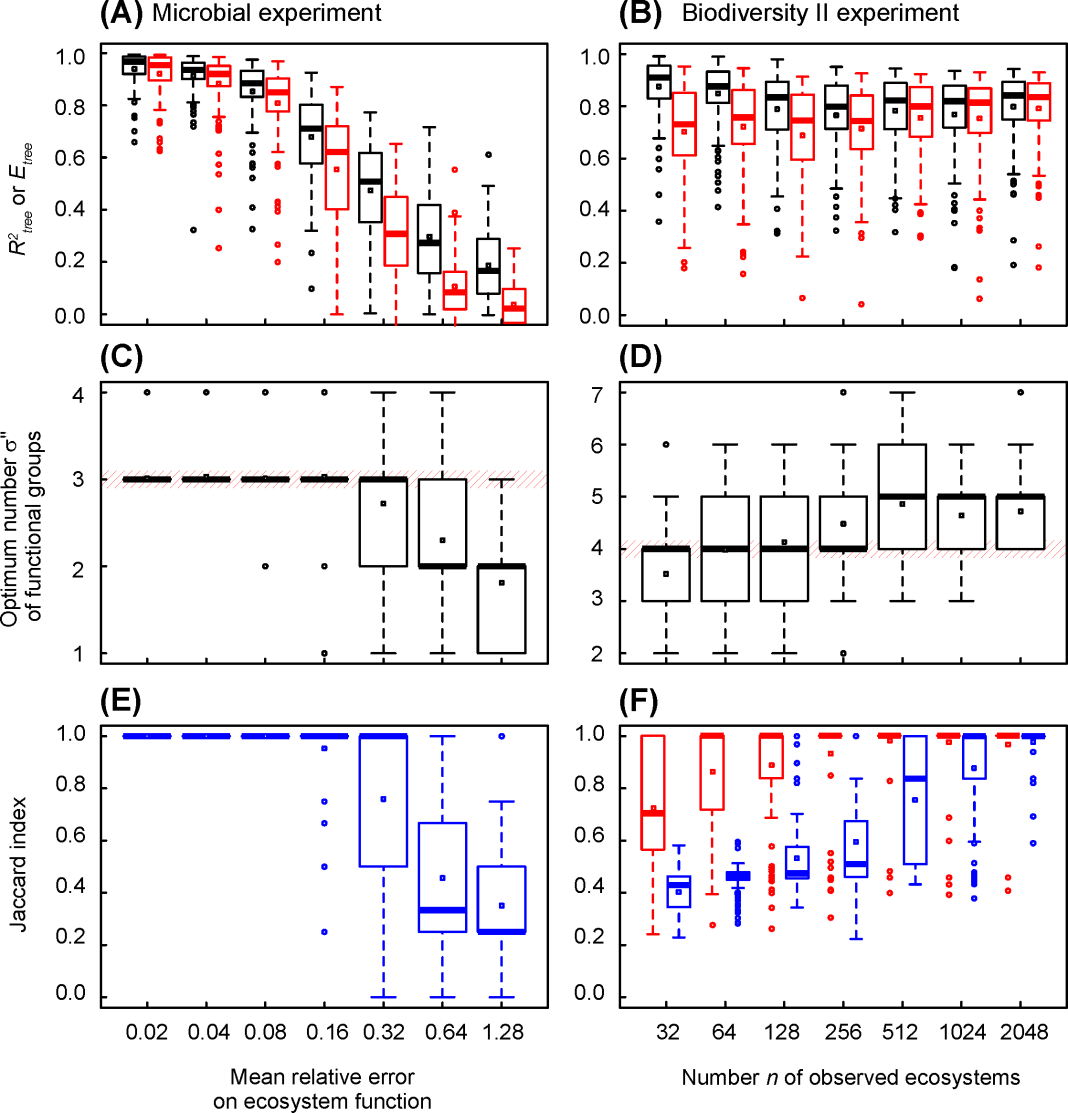
Changes in coefficient of determination, efficiency, optimum number of functional groups and Jaccard index in simulated datasets, versus the number of functional groups of functional structure of ecosystems. The functional structures of ecosystems are those determined by combinatorial analysis of observed datasets (see Fig 2A and 2B), the number of functional groups increasing from the trunk to the leaves of trees. (A), (C) and (E) Simulated dataset mimicking the microbial experiment of Langenheder et al. [16]. All ecosystems are observed and the mean relative error is 0.08. The statistics describe 100 datasets randomly generated. (B), (D) and (F) Simulated dataset mimicking the Biodiversity II experiment of Tilman et al. [17]. The mean relative error is 0.17. The statistics describe 100 random sampling of 2048 ecosystems. (A) and (B) Tree coefficient of determination (in black) and efficiency (in red). (C) and (D) Optimum number of functional groups indicated by tree *AICc*. (E) and (F) Jaccard index.

We first use simulations to test the accuracy of the combinatorial analysis, that is its ability to exactly determine the a priori functional structure of an ecosystem. In the microbial experiment (Fig 4A, 4C and 4E), all the possible ecosystems are simulated and analysed. The Jaccard index is equal to one, except for 5 out of 400 analysed ecosystems: that is less than 1.3% of failure (Fig 4E). The optimum number σ" of functional groups determined using the Akaike index is exact from 2 to 4 functional groups (Fig 4C). It is under-estimated for 5 functional groups. The tree coefficient of determination *R*^*2*^_*tree*_ and tree efficiency *E*_*tree*_ vary in a large range, mainly between 0.3 and 0.9, but the two parameters are close to each other in average: the ratio *E*_*tree*_/*R*^*2*^_*tree*_ is higher than 0.9 from 2 to 4 functional groups (Fig 4A and Figure A in S1 Fig). The results mean that, when the number of functional groups are comprised between 2 and 4, the functional structure of ecosystems is exactly determined by the combinatorial analysis, that is: the number of functional groups, the size of each functional group and the identity of species that belong to each functional group. Moreover, the Akaïke index provides an accurate indication of the true number of functional groups.

The Biodiversity II experiment (Fig 4B, 4D and 4F) confirms this general behaviour. The combinatorial analysis is done on samples containing 2048 ecosystems, out of the 65 519 pluri-species ecosystems. The Jaccard index is equal to one from 2 to 9 functional groups, except for 23 out of 800 analysed ecosystems, that is less than 3.0% of error (Fig 4F). However, the Jaccard index decreases quickly between 10 and 13 functional groups, until reaching zero at 14 and more functional groups. The optimum number of functional groups determined using the Akaïke index is exact in average from 3 to 10 functional groups (Fig 4D). It is over-estimated for 2 functional groups, and under-estimated for more than 10 functional groups. The tree coefficient of determination *R*^*2*^_*tree*_ varies widely, but the tree efficiency *E*_*tree*_ stays close to *R*^*2*^_*tree*_ when the Jaccard index is 1: the ratio *E*_*tree*_/*R*^*2*^_*tree*_ is higher than 0.8 from 2 to 9 functional groups, that is when the functional structure of ecosystems is exactly determined (Fig 4B and Figure B in S1 Fig). The results indicate that a combinatorial analysis can determine, even for a large number of functional groups, that is a complex functional structure of ecosystems, the right number, size and species composition of functional groups of species. Ten functional groups generate 1023 assembly motifs: a sample of 2048 ecosystems is sufficient to determine the right functional structure of ecosystems.

### Effect of relative error and sampling effort on the accuracy of the combinatorial analysis

**Fig 5:**
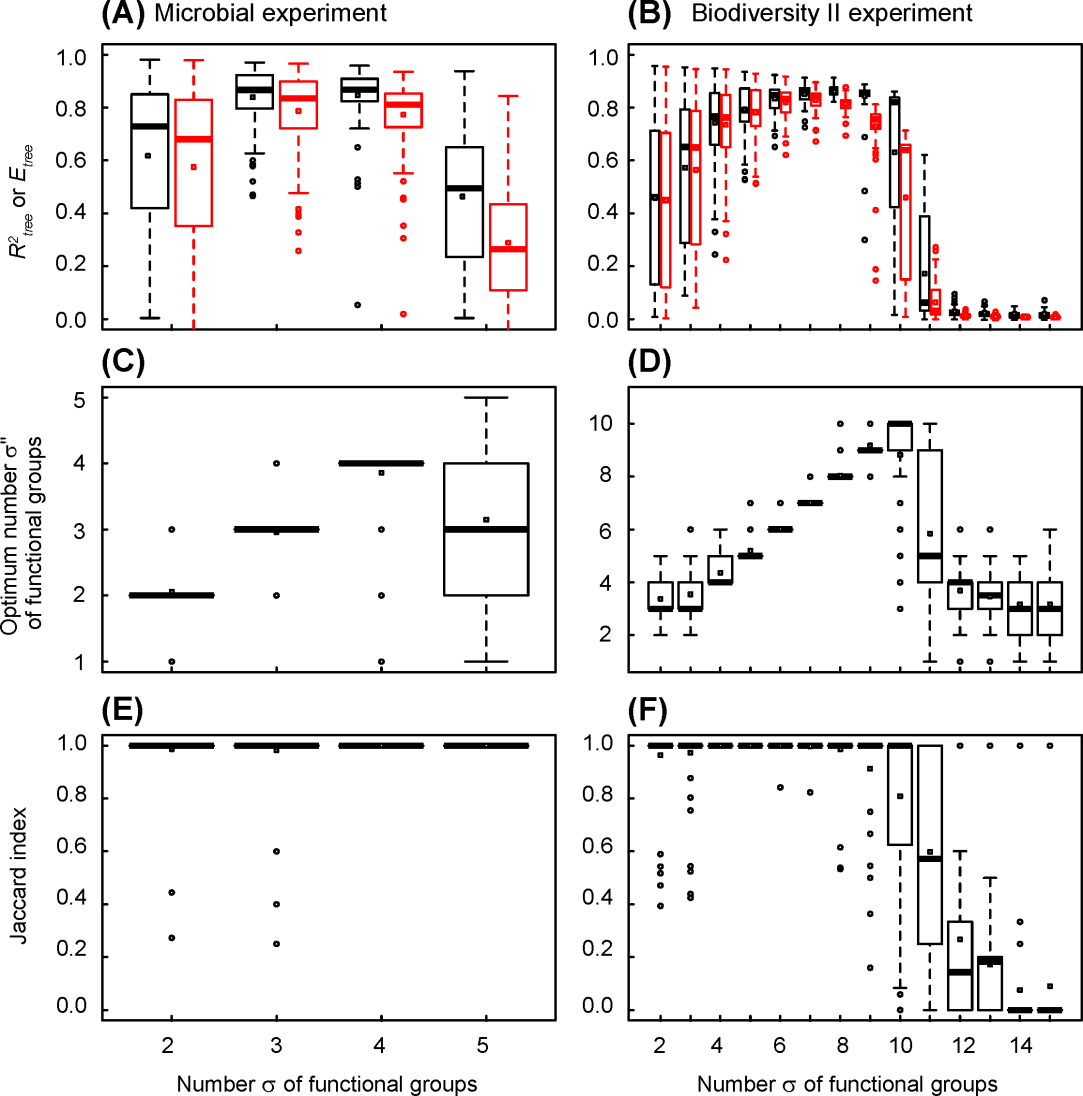
Changes in coefficient of determination, efficiency, optimum number of functional groups and Jaccard index in simulated datasets, versus relative error of ecosystem function and number of observed ecosystems. (A), (C) and (E) Simulated dataset mimicking the microbial experiment of Langenheder et al. [16]. All ecosystems are observed and the mean relative error increases from 0.02 to 1.28. The statistics describe 100 datasets randomly generated. (B), (D) and (F) Simulated dataset mimicking the Biodiversity II experiment of Tilman et al. [17]. The mean relative error is 0.17 and the number of observed ecosystems increases from 32 to 2048. The statistics describe 100 random sampling from a same random dataset. (A) and (B) Tree coefficient of determination (in black) and efficiency (in red). (C) and (D) Optimum number of functional groups indicated by tree *AICc*. (E) and (F) Jaccard index. In blue, by referring to the number of functional groups a priori defined (3 and 4 in microbial and Biodiversity II experiments, respectively). In red, by referring to 3 functional groups in Biodiversity II experiment, resulting from the clustering of the two largest species functional groups.

Next, we use the simulated datasets to test the effect of relative error of observations on the accuracy of species clustering. The tree coefficient of determination *R*^*2*^_*tree*_ is close to 1 when relative error is 2% (Fig 5A). It decreases when relative error increases, and is less than 0.2 when relative error is greater than 100%. The efficiency *E*_*tree*_ follows the same variation: it is less than *R*^*2*^_*tree*_, and the ratio *E*_*tree*_/*R*^*2*^_*tree*_ is higher than 0.8 when relative error is lower than 16% (Figure A in S2 Fig). The optimal number of functional groups is close to 3 (Fig 5C), which is the expected value. The Jaccard index indicates that the 6 species are correctly clustered by the 3 functional groups for relative errors below 32% (Fig 5E).

In the Biodiversity II experiment, only 53 pluri-specific combinations were observed, that is less than 0.1% of all possible assemblages. We use the simulated datasets to test the effect of sampling effort on the accuracy of species clustering. The coefficient of determination decreases slightly with sampling effort (Fig 5B). Conversely, the efficiency increases slightly with sampling effort. The ratio *E*_*tree*_/*R*^*2*^_*tree*_ increases from 0.84 to 0.98 for 32 to 2048 observed ecosystems, respectively (Figure B in S2 Fig). The optimum number of functional groups increases steadily from 3.5 to 4.6 for sampling of 32 to 2048 ecosystems (Fig 5D): it is 4.2 on average over the range of variation, so close to 4 functional groups, which is the expected value.

The Jaccard index, which refers to 4 groups initially defined, indicates that species clustering into 4 functional groups is only right for a sampling effort greater than 512 observed ecosystems, that is about 1% of possible assemblages (Fig 5F). We observe that the species belonging to the smallest clusters, of 1 and 2 species respectively, are always well classified (data not shown). Conversely, the species belonging to the largest clusters, of 6 and 7 species respectively, are often wrongly classified, inducing errors on the size and species composition of clusters. We consequently compute a Jaccard index that refers to 3 functional groups: the two smallest clusters and a larger group that bring together the two largest functional groups (then of 6+7 = 13 species). This index specifies that species clustering into 3 functional groups only is right for a much smaller sampling effort of about 64 observed ecosystems, near 0.1% of all possible assemblages (Fig 5F).

## Discussion

### Identifying the functional structure of ecosystems through a combinatorial analysis of their composition

The combinatorial approach proposed here is based on two assumptions: (i) the function of an ecosystem depends on its species composition, (ii) in a given environment, different species can be functionally redundant, i.e. they can generate similar effects on ecosystem function. The first assumption is the basis of ecology [25]. The second assumption assumes that, at least for the specific function and context under consideration, different species are functionally equivalent [26]. Different but functionally equivalent species can thus be clustered into a single functional effect group (sensu Díaz and Cabido [2]). An assembly motif is a combination of functional groups: it describes the functional composition of an ecosystem sensu Tilman [18]. Each assembly motif is associated with a distinctive level of functioning. In our framework, the assumption of functional redundancy implies that ecosystems that share the same assembly motif, i.e. that are composed of species belonging to the same set of functional groups—whether the species are identical or not—display a similar functioning. Several authors [27–32] have shown that the function of ecosystems can strongly vary with their functional composition. Our findings suggest that this assumption proves to be sufficient to explain and predict an ecosystem function very accurately. More broadly, they highlight the importance of the functional structure of ecosystems in determining their functioning.

Contrary to the method previously presented by Jaillard et al. [14], this method does not require any prior information about the performances of species grown in monoculture. This is a critical improvement, both technically and conceptually. Our novel approach allows to determine the effect of species interactions on ecosystem processes in any natural ecosystem, whatever the number of species, whether the species are known or not. This is possible because our combinatorial method needs species composition and related ecosystem function only as input data. On the other hand, this method cannot quantify the absolute, positive or negative, effects of species interactions. The only known baseline is the mean functioning of the ecosystems sampled. Each assembly motif describes a particular biotic environment. Within this biotic environment, species interactions are unknown, but their effects relative, positive or negative, to the baseline are known. Our combinatorial analysis identifies the functional groups that, through their interactions with other functional groups, shift ecosystem functioning relative to the baseline.

The results obtained with simulated datasets show that our combinatorial approach is very efficient. In the simulated datasets, the effects of species interactions change randomly with the biotic environment: a same species can induce a positive effect when co-occurring with some species, but a negative effect when co-occurring with other species. The ecosystem response is then idiosyncratic, species contributing differently to ecosystem functioning according to the identity of co-occurring species [33]. This is clearly the most difficult scenario to unravel the effects of species interactions [34,35]. Our combinatorial approach does this successfully, whenever observation error is reasonable and sampling effort sufficient [12]. It does it successfully even when the functional structure of ecosystems becomes very complex, with several thousands of different assembly motifs, each motif having in average its own level of functioning.

The effects of biodiversity on ecosystem functioning is likely to be non-linear [13]. Our combinatorial approach does not assume any linearity, which is a critical advantage compared to traditional approaches [6,8,36]. Each species clustering by functional groups is a combinatorial model that captures the non-linear effect of species interactions on ecosystem functioning. This is the reason why the combinatorial approach is so powerful: it is flexible and accounts for all kinds of ecosystem responses, including idiosyncratic responses [10,11]. Each assembly motif describes a particular biotic environment, in which the effects of species interactions are similar. In this homogeneous sub-space, a linear model whose validity is local and restricted can be applied efficiently.

### The accuracy of the combinatorial analysis increases with sampling effort

The novelty of our approach led us to adapt conventional algorithms and statistical tests. This is the case of the coefficient of determination to assess the goodness-of-fit of a clustering tree, and of the efficiency to assess its predictive ability. These statistics characterize the explanatory and predictive capacities, respectively, of a clustering tree of species for all ecosystems, including those whose assembly motifs are poorly represented in the set of sampled ecosystems. Our results show that the coefficient of determination of a clustering model is not a sufficient criterion *per se*. It must be completed by an evaluation of its efficiency: a good model is a model whose efficiency is close to its coefficient of determination, that is a model that predicts as well as it explains the observed functioning by the species composition of ecosystems.

We also set up a method based on Akaïke index to determine the optimal number of functional groups. Our results show that the optimal number of species functional groups is accurate in most of the simulated datasets tested. The correction of second-order bias proposed by Burnham and Anderson [22] limits overfitting for small datasets. This optimal number of functional groups should therefore be considered as an upper limit in order to avoid overfitting of available data. The clustering model that has the optimum number of functional groups is the one that explains the most accurately, but also the most parsimoniously, the deviations of ecosystem functioning from the overall mean. These statistics are essential because they (i) provide a rigorous comparison of different clustering trees by referring to exactly the same ecosystem sample, in number and identity, (ii) determine the optimal number of functional groups, without over- or under-estimating when the sampling size is large enough, and thus (iii) identify the model of species clustering that best captures the ecosystem function.

Our combinatorial approach correctly identifies the hidden functional structure of ecosystems and the identity of species that make up these functional groups. When all or a large part of species combinations are observed and observation error is moderate, the effectiveness of the combinatorial approach is remarkably high: the number and size of functional groups, and the identity of species that compose them, are determined exactly. When a fraction of species combinations are observed, the combinatorial approach is less efficient: it correctly identifies functional groups that contain few species but fails to discriminate within functional groups that contain a large number of species. If the probability of observing different species is uniform, ecosystems associated with assembly motifs composed of large functional groups are the most numerous. Their mean functioning is then necessarily close to the mean functioning of all observed ecosystems, which increases the probability of species to be improperly clustered. It is obviously not possible to observe all possible species assemblages in natura. Our simulations show that, for an ecosystem composed of 16 species only, 64 (respectively 1024) observations are needed to exactly and surely determine 3 (respectively 4) functional groups. In a species-rich ecosystem such as tropical or microbial ecosystem, it is likely that our method will correctly determine with a moderate sampling effort the species or groups of species that strongly change ecosystem functioning. Here we sample ecosystems randomly: it is also likely that the process of ecosystem sampling can be optimized for decreasing the sampling effort.

The probability to correctly cluster the species increases with the sampling effort. Our results show that, with a large sampling effort, the functional structure of an ecosystem is exactly determined, in number and size of functional groups, including the identity of different species that compose each functional group. This result reinforces the role played by the observer's experience [12]. In the absence of previous experience, an observer will generally reduce their analysis to the identification of a few species or species groups which they know as "indicators" of ecosystem functioning. Only a long experience allows them to confidently determine the boundaries of species functional groups whose effects are not very marked.

Wright et al. [36] previously assessed random species clusterings based on their ability to predict an ecosystem function. The authors proceeded by random clustering of species into functional groups. The quality of species clustering was evaluated by the Pearson correlation coefficient of the linear regression between the observed ecosystem function and the number of functional groups, assuming that ecosystem functioning varied mainly and linearly with the functional diversity of ecosystems. The main result obtained by Wright et al. [36] was that the explanatory powers of a priori and random clustering were not significantly different.

Our approach is very different. First, we bypass the combinatorial wall by building a hierarchical tree of species clustering. The number of species clustering possibly constructed using *s* species is given by the Stirling number of the second kind [37]. For instance, 6 and 16 species can be grouped in 203 and 10^11^ possible species clustering, respectively. Our combinatorial approach build the whole tree of species clustering that best accounts for the observed dataset by evaluating at the absolute maximum 50 and 800 species clustering, respectively. Wright et al. [36] note that 34 species allow to construct 10^28^ species clustering: our combinatorial approach would evaluate less than 7106 species clustering.

Second, we use a convergence criterion that does not presuppose any causal relationship. The combinatorial model links each species composition with an ecosystem function: it therefore makes it possible to model the ecosystem function according to its species composition. The convergence is achieved by comparing the modelled functions with the observed functions of ecosystems. Our model does not make other assumptions, in particular no assumption about the shape of ecosystem response to diversity. Our conclusions also are very different from those of Wright et al. [36]: an a posteriori species clustering is highly explanatory and predictive, and allows to identify the species that govern an ecosystem function.

### Ecological implications for the case studies

Langenheder et al.'s (2010) [16] experiment is notable because it tested all the possible combinations of 6 bacteria. The results obtained with our combinatorial approach are likely accurate and fair. Our analysis of this experiment confirms the results previously reported by the authors, especially the major role played by the species SL104 on substrate oxidation. Jaillard et al. [14] showed that substrate oxidation was strongly correlated with the composition effect, but more poorly with the interaction effect. This finding suggests that the species SL104 acts mainly by its own ability to oxidize xylose. The ratio between model efficiency and coefficient of determination is higher than 0.9, meaning that the model predicts a large proportion of the explained variance. The clustering obtained by the combinatorial analysis does not improve the results, likely because all the useful information is already included in the dataset, that holds all the possible bacteria assemblages.

The Biodiversity II Cedar Creek experiment is based on 16 grassland species, of which less than 0.1% of multi-species combinations was observed. The Akaïke index suggests that four functional groups of species can be considered. Tilman et al. [17] showed that plant biomass increases with the number of plant species in this experiment, and thus the effect of species interactions is not idiosyncratic. It is therefore likely that the four functional groups suggested by our analysis correspond to the intrinsic functional structure that really contributes to plant biomass production in the Cedar Creek experiment. The ratio between model efficiency and determination coefficient is higher than 0.8, indicating a high predictive ability of the model. The resulting species clustering highlights the major role played by the legume *Lupinus perennis*. The legume *Lespedeza capitate* appears functionally redundant with the forb *Liatris aspera*. Among the two other functional groups, one group includes the two other legumes, *Petalostemum purpureum* and *Amorpha canescens*, and the C3-grasses. The four functional groups of species account for almost all of the observed variations in plant biomass.

## Conclusions

Our combinatorial approach of ecosystem composition reproduces the associative learning of most people in nature [10,11]. Associative learning is the ancestral way to explore the environment, whether by animals and toddlers, or by any individual immersed in a new environment [38]. The combinatorial approach that we propose is powerful precisely because it requires no a priori knowledge of the constitutive elements of ecosystems, such as species abundances, species functional traits or species interactions. The observer only needs to be able to identify the constitutive elements, species, and to be attentive to their environment [12,39]. The more experienced, the more efficient. Associative learning is descriptive as it does not inform on underlying processes and causal relations. But it makes it possible to identify the critical species or species groups that govern ecosystem functioning. This achievement is the most solid base for building a cognitive, deductive approach of the relationship between biodiversity and ecosystem functioning in the field [40].

## Supporting information

S1 Fig Changes in the ratio efficiency/coefficient of determination versus the number of functional groups of functional structure of ecosystems. The functional structures of ecosystems are those determined by combinatorial analysis of observed datasets (see Fig 2A and 2B), the number of functional groups increasing from the trunk to the leaves of trees. (A) Simulated dataset mimicking the microbial experiment of Langenheder et al. [16]. All ecosystems are observed and the mean relative error is 0.08. The statistics describe 100 datasets randomly generated. (B) Simulated dataset mimicking the Biodiversity II experiment of Tilman et al. [17]. The mean relative error is 0.17. The statistics describe 100 random sampling of 2048 ecosystems.
(TIF)

S2 Fig Changes in in the ratio efficiency/coefficient of determination, versus relative error of ecosystem function and number of observed ecosystems. (A) Simulated dataset mimicking the microbial experiment of Langenheder et al. [16]. All ecosystems are observed and the mean relative error increases from 0.02 to 1.28. The statistics describe 100 datasets randomly generated. (B) Simulated dataset mimicking the Biodiversity II experiment of Tilman et al. [17]. The mean relative error is 0.17 and the number of observed ecosystems increases from 32 to 2048. The statistics describe 100 random sampling from a same random dataset.
(TIF)
