# Correction: A combinatorial analysis using observational data identifies species that govern ecosystem functioning

**DOI:** 10.1371/journal.pone.0203681

**Published:** 2018-09-05

**Authors:** Benoît Jaillard, Philippe Deleporte, Michel Loreau, Cyrille Violle

There is an error in the second sentence of the subsection titled, “Modelling the functioning of an ecosystem based on its species composition” in the Materials and Methods section. The correct sentence is: We consider a sample A of *n* ecosystems *A* observed in the field.

There are errors in the second and third sentences of the subsection titled, “Virtual biodiversity datasets,” in the Materials and Methods section. The correct sentence is: We assume that the function $F(A\in A_{k})$ of each ecosystem is equal to the mean function $\bar{F}(A_{k})$ of ecosystems that share its assembly motif, with a random error $\varepsilon (A\in A_{k})$. The mean functions $\bar{F}(A_{k})$ of ecosystems that share the same assembly motif are randomly drawn inside an interval of values, according to a uniform law.

In the Methods section, there are errors in the first through fifteenth equations. Please view the complete, correct equations here:

In the “Modelling the functioning of an ecosystem based on its species composition,” section:

$$F_{modelled}(A\in A_{k})=\underset{i\in A}{\bar{F}}(A_{i,k})$$

In the “Evaluating the accuracy of a species clustering,” section:

$$TSS(A)=\sum_{A\in A}{\left(F_{observed}(A)-{\bar{F}}_{observed}(A)\right)}^{2}$$

And:

$${RSS}_{modelling}(\sigma ,A)=\sum_{A\in A}{\left(F_{observed}(A)-F_{modelled}(\sigma ,A)\right)}^{2}$$

And:

$$R^{2}(\sigma ,A)=1-\frac{{RSS}_{modelling}(\sigma ,A)}{TSS(A)}$$

In the “Evaluating the predictive ability of a species clustering,” section:

$${RSS}_{predicting}(\sigma ,A)=\sum_{A\in A}{\left(F_{observed}(A)-F_{predicted}(\sigma ,A)\right)}^{2}$$

And:

$$E(\sigma ,A)=1-\frac{{RSS}_{predicting}(\sigma ,A)}{TSS(A)}$$

In the “Building a hierarchical divisive tree of species clustering,” section:

$$\sigma \prime (A)=\underset{j\in [1,\ldots ,\sigma ]}{argmax}(F_{predicted}(j,A))$$

In the “Evaluating the quality of a hierarchical tree of species clustering,” section:

$${RSS}_{tree,modelling}(\sigma ,A)=\sum_{A\in A}{\left(F_{observed}(A)-F_{modelled}(\sigma \prime (A),A)\right)}^{2}$$

And:

$${RSS}_{tree,predicting}(\sigma ,A)=\sum_{A\in A}{\left(F_{observed}(A)-F_{predicted}(\sigma \prime (A),A)\right)}^{2}$$

And:

$$R_{tree}^{2}(\sigma ,A)=1-\frac{{RSS}_{tree,modelling}(\sigma ,A)}{TSS(A)}$$

And:

$$E_{tree}(\sigma ,A)=1-\frac{{RSS}_{tree,predicting}(\sigma ,A)}{TSS(A)}$$

In the “Evaluating the optimum number of functional groups,” section:

$${AICc}_{tree}(\sigma ,A)=n\;log\;\left(\frac{{RSS}_{tree,modelling}(\sigma ,A)}{n}\right)+2m+\frac{2m(m+1)}{\left(n-m+1\right)}.$$

And:

$$\sigma "(A)=\underset{\sigma \in [1,\ldots ,s]}{argmin}({AICc}_{tree}(\sigma ,A))$$

In the “Virtual biodiversity datasets,” section:

$$F(A\in A_{k})=\bar{F}(A_{k})+\varepsilon (A\in A_{k})$$

And:

$$\varepsilon (A\in A_{k})∼\bar{F}(A_{k})\;N(0,1)c_{v}$$

The images for Figs 4 and 5 are incorrectly switched. The image that appears as Fig 4 should be Fig 5, and the image that appears as Fig 5 should be Fig 4. The figure captions appear in the correct order.

**Fig 4:**
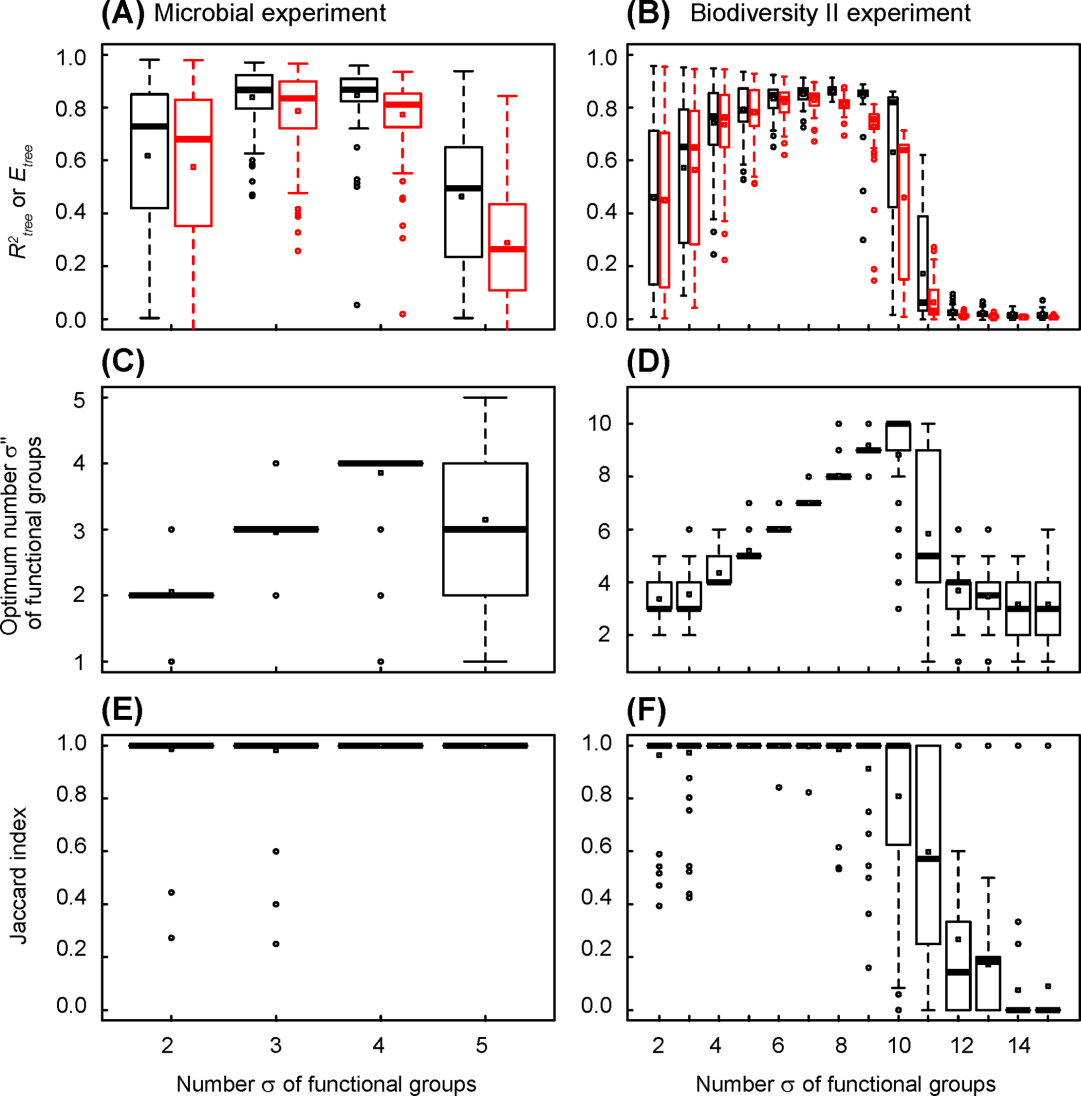
Changes in coefficient of determination, efficiency, optimum number of functional groups and Jaccard index in simulated datasets, versus the number of functional groups of functional structure of ecosystems. The functional structures of ecosystems are those determined by combinatorial analysis of observed datasets (see Fig 2A and 2B), the number of functional groups increasing from the trunk to the leaves of trees. (A), (C) and (E) Simulated dataset mimicking the microbial experiment of Langenheder et al. [16]. All ecosystems are observed and the mean relative error is 0.08. The statistics describe 100 datasets randomly generated. (B), (D) and (F) Simulated dataset mimicking the Biodiversity II experiment of Tilman et al. [17]. The mean relative error is 0.17. The statistics describe 100 random sampling of 2048 ecosystems. (A) and (B) Tree coefficient of determination (in black) and efficiency (in red). (C) and (D) Optimum number of functional groups indicated by tree AICc. (E) and (F) Jaccard index.

**Fig 5:**
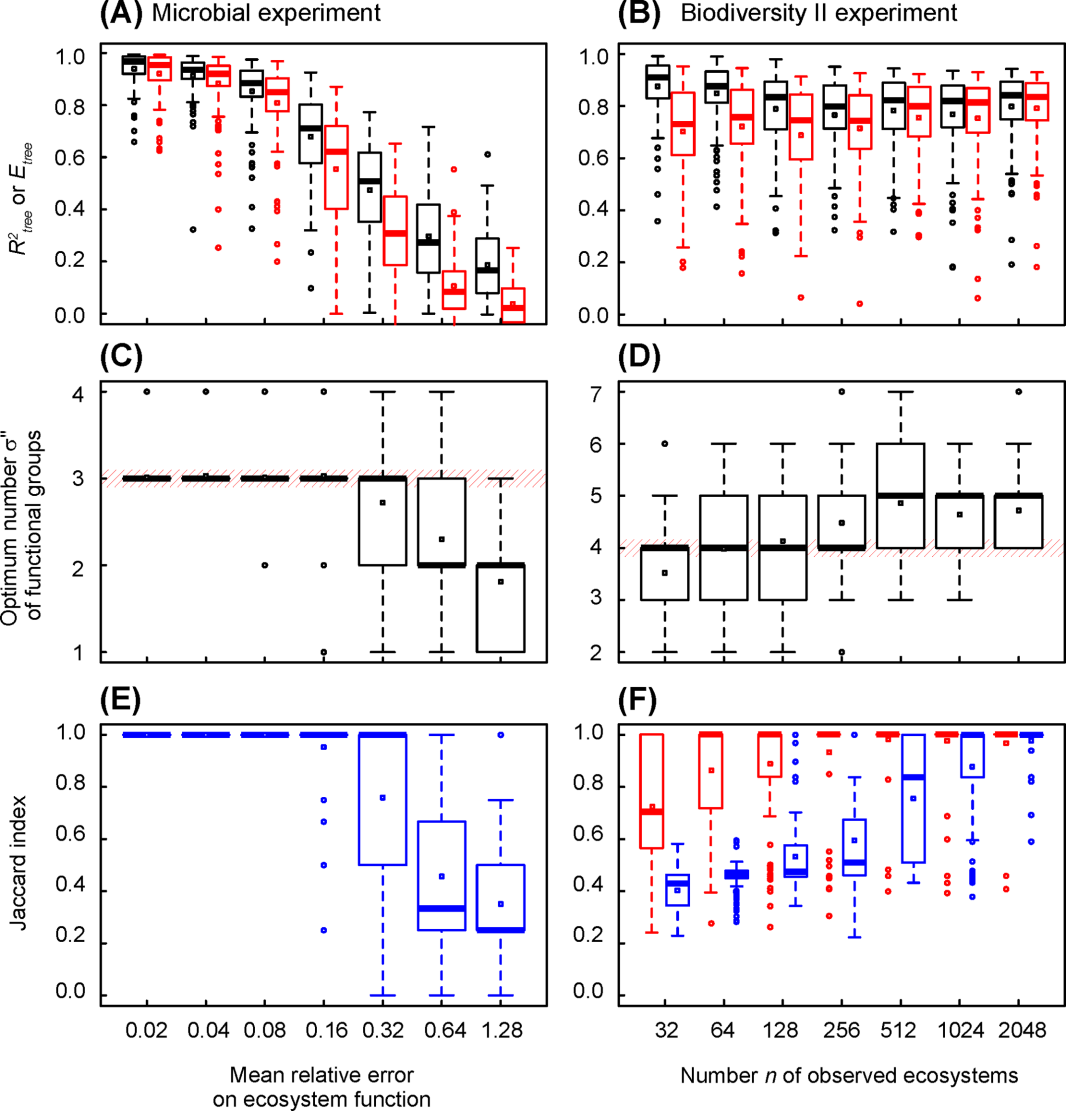
Changes in coefficient of determination, efficiency, optimum number of functional groups and Jaccard index in simulated datasets, versus relative error of ecosystem function and number of observed ecosystems. (A), (C) and (E) Simulated dataset mimicking the microbial experiment of Langenheder et al. [16]. All ecosystems are observed and the mean relative error increases from 0.02 to 1.28. The statistics describe 100 datasets randomly generated. (B), (D) and (F) Simulated dataset mimicking the Biodiversity II experiment of Tilman et al. [17]. The mean relative error is 0.17 and the number of observed ecosystems increases from 32 to 2048. The statistics describe 100 random sampling from a same random dataset. (A) and (B) Tree coefficient of determination (in black) and efficiency (in red). (C) and (D) Optimum number of functional groups indicated by tree AICc. (E) and (F) Jaccard index. In blue, by referring to the number of functional groups a priori defined (3 and 4 in microbial and Biodiversity II experiments, respectively). In red, by referring to 3 functional groups in Biodiversity II experiment, resulting from the clustering of the two largest species functional groups.
