# Supplementary figures and images for: A combinatorial analysis using observational data identifies species that govern ecosystem functioning
Source: PLoS One. 2018 Aug 1;13(8):e0201135. doi: 10.1371/journal.pone.0201135 (PMC6070253; doi:10.1371/journal.pone.0201135)

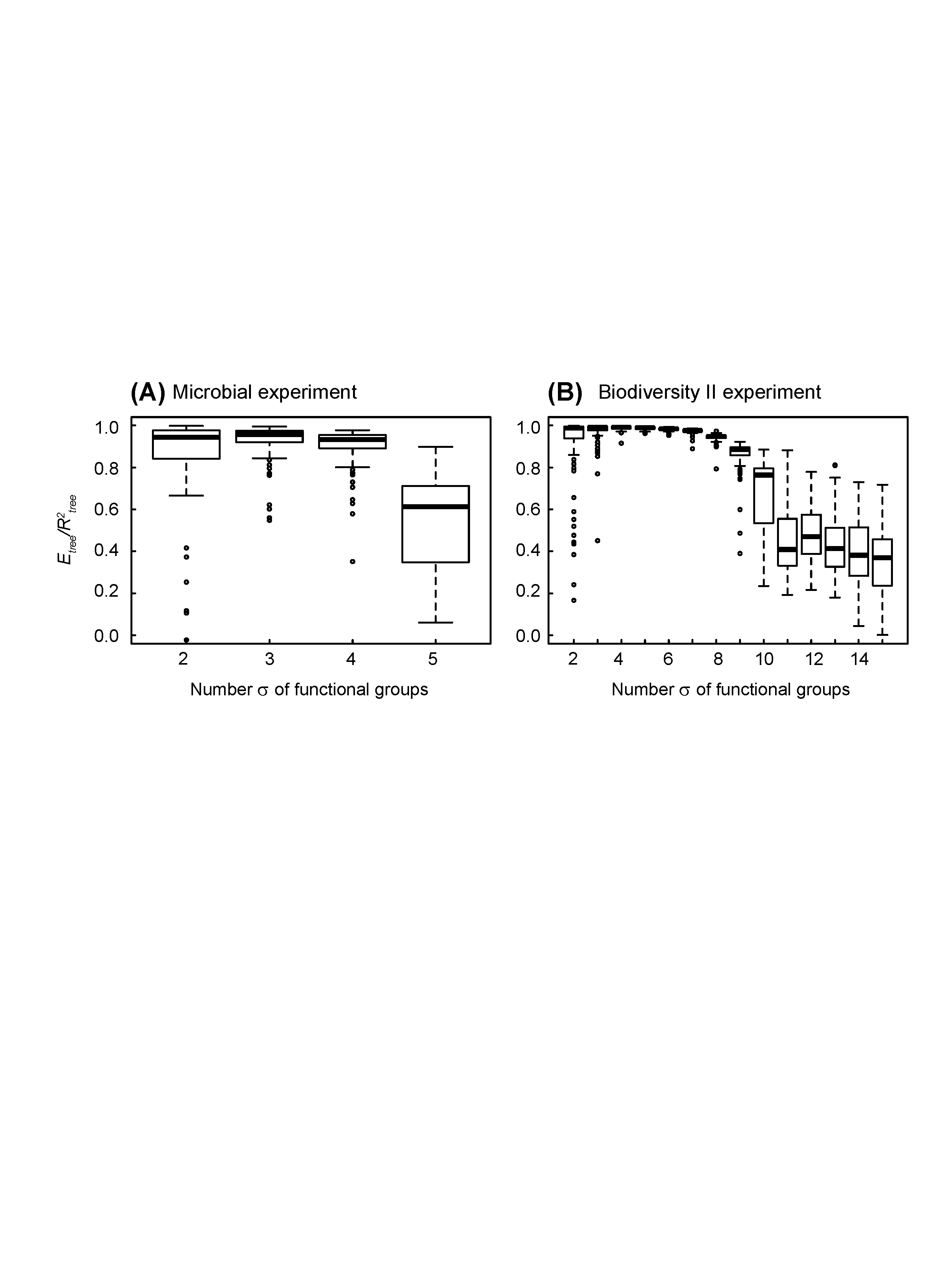

Supplement: S1 Fig — The functional structures of ecosystems are those determined by combinatorial analysis of observed datasets (see Fig 2A and 2B), the number of functional groups increasing from the trunk to the leaves of trees. (A) Simulated dataset mimicking the microbial experiment of Langenheder et al. [16]. All ecosystems are observed and the mean relative error is 0.08. The statistics describe 100 datasets randomly generated. (B) Simulated dataset mimicking the Biodiversity II experiment of Tilman et al. [17]. The mean relative error is 0.17. The statistics describe 100 random sampling of 2048 ecosystems. (TIF) [file pone.0201135.s001.tif]

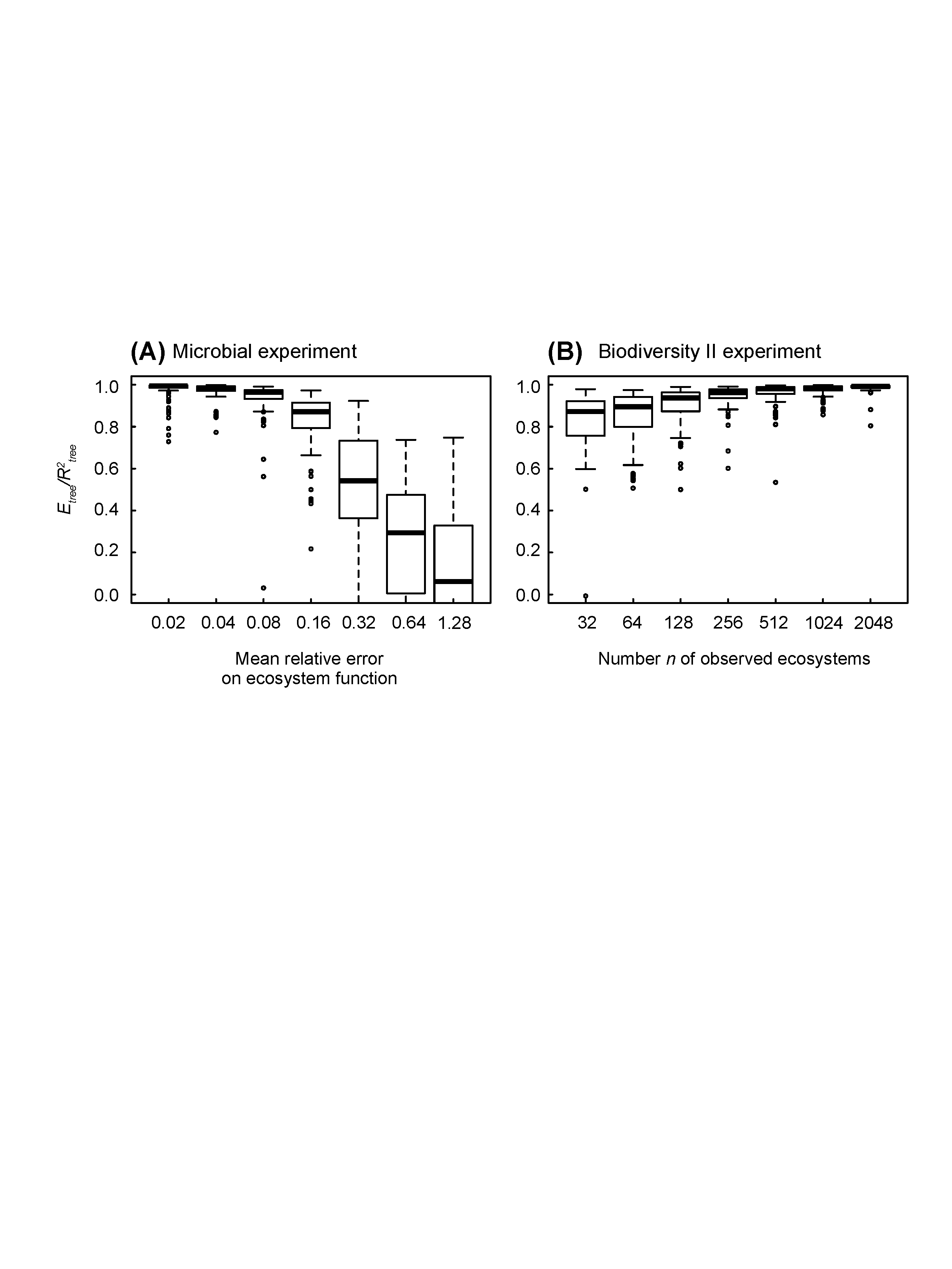

Supplement: S2 Fig — (A) Simulated dataset mimicking the microbial experiment of Langenheder et al. [16]. All ecosystems are observed and the mean relative error increases from 0.02 to 1.28. The statistics describe 100 datasets randomly generated. (B) Simulated dataset mimicking the Biodiversity II experiment of Tilman et al. [17]. The mean relative error is 0.17 and the number of observed ecosystems increases from 32 to 2048. The statistics describe 100 random sampling from a same random dataset. (TIF) [file pone.0201135.s002.tif]
